# Arts and culture engagement and mortality: A population-based prospective cohort study

**DOI:** 10.1177/14034948231165853

**Published:** 2023-04-22

**Authors:** Anita Jensen, Mirnabi Pirouzifard, Martin Lindström

**Affiliations:** Social Medicine and Health Policy, Department of Clinical Sciences and Centre for Primary Health Care Research, Lund University and Region Skåne, Sweden

**Keywords:** Arts and culture activities, visit to theatre/cinema, visit to arts exhibition, social participation, mortality, cardiovascular mortality, cancer mortality, public health, Sweden

## Abstract

**Aims:**

The aim of this study was to investigate associations between having visited the theatre/cinema and an arts exhibition during the past year and all-cause, cardiovascular disease (CVD), cancer and other-cause mortality.

**Methods:**

The 2008 public health postal survey in Scania, Sweden, was distributed to a stratified random sample of the adult population (18–80 years old). The participation rate was 54.1%, and 25,420 participants were included in the present study. The baseline 2008 survey data were linked to cause-of-death register data to create a prospective cohort with 8.3-year follow-up. Associations between visit to the theatre/cinema, visit to an arts exhibition and mortality were investigated in survival (Cox) regression models.

**Results:**

Just over a quarter (26.5%) had visited both the theatre/cinema and an arts exhibition during the past year, 36.6% only the theatre/cinema, 4.9% only an arts exhibition and 32% neither of the two. Not visiting the theatre/cinema during the past year was associated with higher all-cause and CVD mortality. Not visiting an arts exhibition was associated with higher all-cause and other-cause mortality. The combination of having visited neither the theatre/cinema nor an arts exhibition during the past year was associated with higher all-cause, CVD and other-cause mortality.

**Conclusions:**

**There is an association between attending arts and culture activities and a reduced risk of CVD and other-cause mortality but not cancer mortality, although model imperfections are possible.**

## Introduction

Non-communicable diseases explain approximately 71% of global mortality according to the World Health Organization [1]. In 2019, an estimated 17.9 million people died from cardiovascular diseases (CVDs), representing 32% of all global deaths, and 38% of 17 million premature deaths (below the age of 70) were caused by CVD [2]. Although CVDs are highly preventable, they are the most common cause of death in the world [3]. CVDs can be prevented by addressing behavioural risk factors (including tobacco use, unhealthy diet and obesity, physical inactivity and harmful use of alcohol) [2]. They place a considerable burden on budgets and health-care systems [4].

In Sweden, CVD is the main cause of morbidity and mortality [5]. Measures to reduce the burden of CVD have been prioritised in Sweden with initiatives and programmes implemented by the Swedish National Institute of Public Health [6]. Such initiatives and programmes are combinations of both community- and individual-based strategies [5]. Cancer is a leading cause of death worldwide in countries of all income levels, and cases and deaths are expected to rise rapidly as populations grow, age and adapt lifestyles that increase cancer risk [7]. In the Nordic countries, more than one in four deaths were due to cancer in 2015, and cancer is the disease group causing the most burden in terms of disability-adjusted life years (DALYs), to a higher extent even than CVDs [8].

Using the arts for health promotion and prevention of illness has increased on a global scale [9] as well as in Scandinavia [10]. Epidemiological studies have shown that engagement with the arts and culture can have positive effects on physical health. Arts and culture engagement may protect against chronic pain in old age [11]. Such engagement has also shown associations with survival [12] (and longevity with prolonged follow-up time [13]) and a lower risk of CVD and cancer mortality [14] and cancer incidence in urban areas [15]. They can also have mental health benefits such as increased life satisfaction and a lower risk of anxiety and depression [16]. Still, only a few prospective cohort studies, including the Norwegian HUNT study [14], are available that look at associations between arts and culture engagement and mortality.

The aim of this study was to investigate associations between having visited the theatre/cinema and an arts exhibition during the past year and all-cause, CVD, cancer and other-cause mortality in a prospective cohort study, adjusting for relevant covariates.

## Methods

### Study population

A public health survey was conducted in Scania, southern Sweden, in the autumn of 2008. This cross-sectional survey was based on a stratified sample of the adult (between 18 and 80 years old) register population on 1 January 2008. A letter of invitation was posted together with a questionnaire, and three postal reminders were sent out to the sample population. A questionnaire version was also available online. Some 28,198 respondents chose to participate (a 54.1% participation rate). This cross-sectional public health survey was conducted by Region Skåne, the regional public authority responsible for the health-care system in Scania. The public health questionnaire included 134 items including, for example, socio-demographic characteristics, self-reported health, self-reported psychological health (e.g. GHQ12, SF36), social support, social capital, working conditions, health-related behaviours, discrimination and items related to neighbourhood security. The public health questionnaire in Scania 2008 was designed to achieve a broad general overview of the public health situation in Scania at that point in time. The stratified random sample was stratified geographically by 59 municipalities and city parts (in four major cities; Malmö, Lund, Helsingborg and Kristianstad). The number of participants in this geographical stratification was based on age, sex and education to obtain statistical power in all areas. The stratified sample was selected by Statistics Sweden from the national population register where the population weight was created. The weight compensates for the stratification to achieve representativeness of the entire Scania population. The 2008 cross-sectional baseline data were linked to register-based mortality (causes of death register) from the National Board of Health and Welfare (Dödsorsaksregistret at Socialstyrelsen), creating a prospective closed cohort population.

### Dependent variable

All-cause and diagnosis-specific mortality was followed prospectively from between 27 August and 14 November 2008 (depending on registration date of the individual answer) to 31 December 2016 (8.3 years onwards), or until death. A total of 25,420 participants were included in this study, after exclusion of 2642 respondents with internally missing values on any or several of the items analysed in this study. A further 136 participants in 2008 were lost at the follow-up. All respondents with any (one or more) internally missing values on any of the items/variables included in the multiple survival (Cox) regression analyses were thus excluded. The International Classification of Diseases 10 was used for causes of death. The connection of baseline survey data with national death register data is possible by using the 10-digit person number system. This was conducted by a third party (private company). The person numbers were erased from the data set before delivery from the National Board of Health and Welfare to the research group.

All-cause (total), CVD (I00–I98), cancer (C00–C97) and all other causes (causes other than I00–I98 and C00–C97) of mortality were included as broad categories. All-cause mortality is the sum of the three broad cause-specific categories above.

### Independent variables

The two items ‘visit to the theatre/cinema during the past year’ and ‘visit to an arts exhibition during the past year’ were assessed with yes or no responses. The phrasing ‘past year’ refers to the period from autumn 2007 to autumn 2008 (depending on the exact date the respondent answered the questionnaire in the autumn of 2008). In Tables I and II, these two items were combined into the four categories: (a) visited both activities during the past year, (b) visited only the theatre/cinema, (c) visited only an arts exhibition and (d) none of the two (no activity). The two items are part of a larger social participation category item which includes 13 items as well as the option to report none of the 13 activities during the past year at all. Theatre/cinema and arts exhibition were extracted because they are the only two items among the 13 social participation items that depict cultural activities.

**Table I.** Descriptive characteristics (%) of age, sex, SES, country of birth, chronic disease, low leisure-time physical activity, smoking, alcohol consumption and generalised trust in other people by social participation.

	(Social participation)				*p*-Value
	Both theatre/cinema and arts exhibition	Only theatre/cinema	Only arts exhibition	Neither theatre/cinema nor arts exhibition	
	*N*=7036	*N*=8938	*N*=1350	*N*=8096	
	26.5%	36.6%	4.9%	32%	
Age, years (*M*±*SD*)^a^	46.1±16.3 (45.6–46.6)	40.3±15.9 (39.9–40.7)	54.4±15.0 (53.3–55.5)	50.8±16.4 (50.4–51.3)	<0.001
Sex^b^					<0.001
Male	44.4 (43.0–45.9)	47.1 (45.7–48.4)	53.2 (49.9–56.5)	56.9 (55.6–58.3)	
Female	55.6 (54.1–57)	52.9 (51.6–54.3)	46.8 (43.5–50.1)	43.1 (41.7–44.4)	
SES^b^					<0.001
High non-manual	16.5 (15.4–17.5)	8.3 (7.7–9.0)	8.7 (7.0–10.5)	3.4 (3.0–3.9)	
Medium non-manual	20.1 (19.0–21.2)	15.5 (14.6–16.4)	14.0 (11.7–16.3)	7.1 (6.5–7.8)	
Low non-manual	7.6 (6.8–8.3)	10.0 (9.2–10.7)	7.0 (5.2–8.7)	6.1 (5.4–6.8)	
Skilled manual	5.8 (5.1–6.5)	13.3 (12.4–14.2)	7.1 (5.3–8.8)	11.9 (11.0–12.8)	
Unskilled manual	7.3 (6.5–8.1)	14.5 (13.6–15.4)	6.7 (5.1–8.3)	15.7 (14.7–16.7)	
Self-employed/farmer	6.8 (6.1–7.5)	5.9 (5.3–6.5)	6.6 (4.8–8.3)	5.6 (4.9–6.2)	
Early retired	1.9 (1.5–2.3)	1.9 (1.6–2.2)	5.3 (3.8–6.8)	7.4 (6.6–8.1)	
Unemployed	2.7 (2.1–3.2)	3.6 (3.1–4.1)	2.2 (1.1–3.3)	5.8 (5.0–6.5)	
Student	10.1 (9.1–11.1)	10.3 (9.4–11.2)	4.1 (2.5–5.8)	5.4 (4.6–6.1)	
Old-age pensioner	16.8 (15.8–17.8)	9.1 (8.5–9.7)	32.4 (29.4–35.4)	24.8 (23.7–25.8)	
Unclassified	4.1 (3.4–4.8)	6.9 (6.1–7.6)	4.4 (2.7–6.1)	4.9 (4.2–5.6)	
Long-term sick leave	0.5 (0.3–0.7)	0.8 (0.6–1.0)	1.5 (0.6–2.4)	2.0 (1.6–2.4)	
Country of birth^b^	12.5 (11.4–13.6)	14.5 (13.5–15.6)	17.4 (14.6–20.3)	25.7 (24.4–27.1)	<0.001
Chronic disease^b^	24.8 (23.5–26.0)	23.7 (22.6–24.8)	34.6 (31.4–37.7)	36.0 (34.7–37.4)	<0.001
Low leisure-time physical activity^b^	6.3 (5.5–7.1)	12.0 (11.1–12.8)	12.3 (10.1–14.5)	22.5 (21.3–23.7)	<0.001
Tobacco smoking^b^					<0.001
Daily	8.1 (7.2–8.9)	12.5 (11.6–13.4)	14.6 (12.0–17.2)	21.5 (20.4–22.6)	
Yes, but not daily	6.1 (5.4–6.8)	5.4 (4.8–6.0)	5.7 (3.9–7.4)	4.1 (3.5–4.6)	
No	85.8 (84.8–86.9)	82.1 (81.1–83.2)	79.8 (76.7–82.8)	74.4 (73.2–75.6)	
Alcohol drinking past year^b^					<0.001
Never	4.9 (4.2–5.6)	8.4 (7.6–9.2)	10.0 (8.0–12.0)	20.7 (19.5–21.9)	
Once a month or more seldom	14.3 (13.3–15.3)	25.3 (24.2–26.4)	20.2 (17.3–23.0)	27.7 (26.4–29.0)	
2–4 times a month	37.8 (36.4–39.2)	42.0 (40.7–43.3)	27.3 (24.3–30.4)	28.9 (27.6–30.1)	
2–3 times a week	32.5 (31.2–33.8)	20.2 (19.2–21.2)	28.9 (25.7–32.1)	15.7 (14.7–16.7)	
At least 4 times a week	10.5 (9.7–11.3)	4.1 (3.6–4.5)	13.6 (11.4–15.8)	7.1 (6.4–7.8)	
Generalised trust in other people^b^					<0.001
Low	26.3 (24.9–27.6)	37.4 (36.2–38.7)	31.5 (28.5–34.5)	45.5 (44.1–46.9)	

The 2008–2016 Public Health Survey of Scania, Sweden. Total population *N*=25,420. Weighted prevalence. The values in parentheses are 95% confidence intervals for mean or percent based on bootstrap method with 1000 replicates.

a *p*-Value: independent samples ANOVA-test, two-tailed.

b *p*-Value: Pearson chi-square test, two-sided.

SES: socio-economic status; ANOVA: analysis of variance.

**Table II.** Crude model: HRRs with 95% CIs of all-cause mortality.

		Crude		Number of deaths
Cause of death	Ref.	HRR	(95% CI)	
All causes				1304
Theatre or cinema	Active	3.5***	(3.1–4.1)	
Arts exhibition	Active	1.4***	(1.2–1.7)	
Sex	Male	0.6***	(0.5–0.6)	
Age		1.1***	(1.1–1.1)	
SES	High non-manual			
Medium non-manual		0.9	(0.5–1.7)	
Low non-manual		1.2	(0.6–2.4)	
Skilled manual		1.2	(0.7–2.3)	
Unskilled manual		1.0	(0.5–1.9)	
Self-employed/farmer		1.2	(0.6–2.4)	
Early retired		10.9***	(6.3–19.0)	
Unemployed		1.4	(0.6–3.1)	
Student		0.2*	(0.1–0.7)	
Old-age pensioner		17.4***	(10.5–28.6)	
Unclassified		0.6	(0.2–2.0)	
Long-term sick leave		9.5***	(4.8–18.6)	
Country of birth	Swedish	0.9	(0.7–1.0)	
Chronic disease	No	3.2***	(2.8–3.6)	
Leisure-time physical activity	Active	2.7***	(2.4–3.1)	
Tobacco smoking	No			
Daily		1.5***	(1.3–1.8)	
Yes, but not daily		0.6*	(0.4–0.9)	
Alcohol drinking past year	Never			
Once a month or more seldom		0.7**	(0.6–0.9)	
2–4 times a month		0.4***	(0.3–0.4)	
2–3 times a week		0.5***	(0.4–0.7)	
At least 4 times a week		1.2	(1.0–1.6)	
Generalised trust in other people	No	0.9	(0.8–1.1)	

The 2008–2016 Scania Public Health Survey with 8.3-year follow-up, men and women combined. Total population *N*=25,420. Weighted prevalence. Weighted hazard ratios. Bootstrap method (1000 replicates) for variation estimation. Statistically significant values are shown in bold.

* *p*<0.05; **p<0.01; ***p<0.001.

HRR: hazard rate ratio; 95% CI: 95% confidence interval.

With regards to sex, men and women were collapsed in all analyses, and age was included in the analyses as a continuous variable. Country of birth was defined as either born in Sweden (see Table I) or born in any country other than Sweden.

Socio-economic status (SES; by occupation and in relation to the labour market) is divided by Statistics Sweden into non-manual employees in higher, medium and lower positions; skilled and unskilled manual workers; and the self-employed (including farmers). The categories outside the workforce comprise the unemployed (job seekers) as well as the ‘not job seeking’ categories such as those who have retired early (<65 years of age), old-age pensioners (>65 years of age), students and people on long-term sick leave. There is also an unclassified category.

Information regarding chronic disease was obtained by the item ‘Do you have any long-term disease, ailment or injury, any disability or other weakness?’ with response options of yes and no (Table I). Smoking was measured with the item ‘Do you smoke?’ with response options of daily, non-daily and non-smoker (Table I). Leisure-time physical activity (LTPA) was obtained with four alternatives: (a) regular exercise (at least three times per week for at least 30 minutes/occasion, leading to sweating), (b) moderate regular exercise (exercising once or twice per week for at least 30 minutes/occasion, leading to sweating), (c) moderate exercise (more than two hours walking, cycling or equivalent activity/week) and (d) low or no LTPA (less than two hours walking, cycling or equivalent activity/week). The first three alternatives were collapsed as high LTPA (Table I) and the fourth as low LTPA. The LTPA variable in the 2008 survey has been described previously [17].

Alcohol consumption during the past year was measured with the item ‘How often have you consumed alcohol during the past 12 months?’ with response options of daily or almost daily, several occasions per week, once per week, two or three times per month, once per month, once or a few times per half year and more seldom or never.

Generalised trust in other people is self-reported and reflects self-perceived trust. The statement ‘Generally, you can trust other people’, with four response options of ‘do not agree at all’, ‘do not agree’, ‘agree’ and ‘completely agree’, was dichotomised, with the two first options indicating low trust and the two latter indicating high trust (Table I).

### Statistics

**Figure 1.**
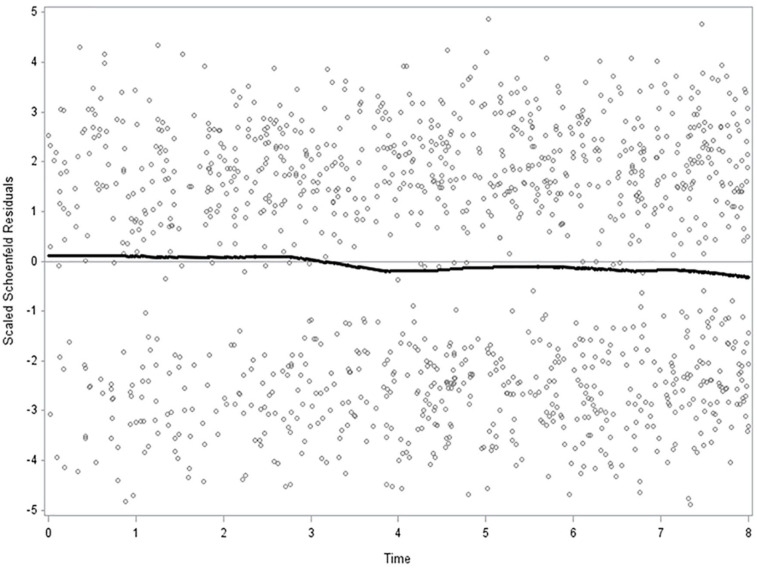
Schoenfeld residuals according to active versus not active and all-cause mortality over the 8.3-year period. The 2008–2016 Scania Public Health Survey with 8.3-year follow-up, men and women combined. *N*=25,420. The proportionality test with interaction term between active versus not active (categories 1, 2 and 3, *n*=17,324, versus category 4, *n*=8096) and all-cause mortality over the 8.3-year period is not significant (*p*=0.101) which indicates proportionality).

Prevalence (%) of all variables stratified by participation/visiting both the theatre/cinema and an arts exhibition, only the theatre/cinema, only an arts exhibition or neither the theatre/cinema nor an arts exhibition during the past year were calculated. The differences between these four categories of participation were assessed using an analysis of variance test for continuous variables and chi-square test for categorical variables (*p*-values; Table I). Associations between each item/variable included in this study and all-cause mortality were analysed in univariate survival analyses with hazard rate ratios (HRRs) and 95% confidence intervals (95% CI; Table II). HRRs with 95% CIs for associations between visits to the theatre/cinema or no visit to the theatre/cinema during the past year and all-cause, CVD, cancer and other-cause mortality were calculated in multiple adjusted models. Five models were calculated: model 0 unadjusted; model 1 adjusted for sex and age; model 2 adjusted for sex, age, country of birth, SES and chronic disease; model 3 additionally adjusted for smoking, leisure-time physical activity and alcohol consumption; and model 4 additionally adjusted for generalised trust in other people (Table III). HRRs with 95% CIs for associations between visits to an arts exhibition or no visit to an arts exhibition during the past year and all-cause, CVD, cancer and other-cause mortality were calculated in multiple adjusted models (Table IV) with corresponding models 0–4 as for visits to the theatre/cinema (Table III). Finally, the theatre/cinema and arts exhibition items were combined and analysed with the groups having visited both the theatre/cinema and an arts exhibition, only the theatre/cinema and only an arts exhibition in the past year in one category (active), and the group who visited neither the theatre/cinema nor an arts exhibition in the past year (not active) in the other category. The active and not active categories were analysed according to all-cause, CVD, cancer and other mortality in multiple adjusted survival models (Table V) with corresponding models 0–4 as in Table III. Follow-up time (days) was included from baseline to death or last follow-up date (31 December 2016). Analysis of sampling variability without distributional assumptions regarding the study population was made possible by bootstrap analysis (SAS/STAT Software Survey Analysis, 2021). Variance estimation on weighted data with confidence intervals and *p*-values were calculated accurately with bootstrap analyses including 1000 replicates. The assumption of proportional hazards was determined by introducing an interaction term with time and visit to the theatre/cinema and an arts exhibition, respectively, during the past year to test the assumption of proportional hazards. Schoenfeld residuals were calculated for theatre/cinema during the past year and arts exhibition during the past year (active versus not active) and mortality (Figure 1). Calculations were performed using SAS v9.4 (SAS Institute, Cary, NC).

**Table III.** HRRs with 95% CIs of all-cause, CVD, cancer and other-cause mortality according to theatre/cinema visit at least once during the past year.

	Model 0		Model 1		Model 2		Model 3		Model 4		Number of deaths
Cause of death	HR	(95% CI)	HR	(95% CI)	HR	(95% CI)	HR	(95% CI)	HR	(95% CI)	
All causes											1304
Theatre/cinema visit	1.0		1.0		1.0		1.0		1.0		
No visit	3.5***	(3.1–4.0)	1.8***	(1.6–2.1)	1.6***	(1.4–1.9)	1.3**	(1.1–1.5)	1.3**	(1.1–1.5)	
Cardiovascular disease											383
Theatre/cinema visit	1.0		1.0		1.0		1.0		1.0		
No visit	4.9***	(3.7–6.3)	2.3***	(1.7–3.0)	2.1***	(1.6–2.7)	1.5**	(1.1–2.1)	1.5**	(1.1–2.1)	
Cancer											517
Theatre/cinema visit	1.0		1.0		1.0		1.0		1.0		
No visit	2.5***	(2.0–3.1)	1.3*	(1.1–1.7)	1.3*	(1.0–1.6)	1.1	(0.9–1.4)	1.1	(0.9–1.4)	
Others											404
Theatre/cinema visit	1.0		1.0		1.0		1.0		1.0		
No visit	4.1***	(3.2–5.3)	2.2***	(1.7–2.8)	1.8***	(1.4–2.4)	1.3	(1.0–1.8)	1.3	(1.0–1.8)	

The 2008–2016 Scania Public Health Survey with 8.3-year follow-up, men and women combined. Weighted hazard ratios. Bootstrap method (1000 replicates) for variation estimation. Model 0 unadjusted; model 1 adjusted for sex and age; model 2 additionally adjusted for socioeconomic status, country of birth and chronic disease; model 3 additionally adjusted for leisure-time physical activity, smoking and alcohol consumption; model 4 additionally adjusted for generalised trust in other people. Statistically significant values are shown in bold.

* *p*<0.05; **p<0.01; ***p<0.001.

**Table IV.** HRRs with 95% CIs of all-cause, CVD, cancer and other-cause mortality according to arts exhibition visit at least once during the past year.

Cause of death	Model 0		Model 1		Model 2		Model 3		Model 4		Number of deaths
	HR	(95%CI)	HR	(95%CI)	HR	(95%CI)	HR	(95%CI)	HR	(95% CI)	
All causes											1304
Arts exhibition visit	1.0		1.0		1.0		1.0		1.0		
No visit	1.5***	(1.2–1.7)	1.6***	(1.4–1.9)	1.6***	(1.3–1.8)	1.3**	(1.1–1.5)	1.3**	(1.1–1.5)	
Cardiovascular disease											383
Arts exhibition visit	1.0		1.0		1.0		1.0		1.0		
No visit	1.5**	(1.2–2.0)	1.7***	(1.2–2.2)	1.6**	(1.2–2.1)	1.2	(0.9–1.6)	1.2	(0.9–1.6)	
Cancer											517
Arts exhibition visit	1.0		1.0		1.0		1.0		1.0		
No visit	1.1	(0.9–1.5)	1.3*	(1.0–1.7)	1.3*	(1.0–1.6)	1.2	(0.9–1.5)	1.2	(0.9–1.5)	
Others											404
Arts exhibition visit	1.0		1.0		1.0		1.0		1.0		
No visit	2.0***	(1.5–2.6)	2.2***	(1.7–2.9)	2.0***	(1.5–2.7)	1.6**	(1.2–2.2)	1.6**	(1.2–2.2)	

The 2008–2016 Scania Public Health Survey with 8.3-year follow-up, men and women combined. Weighted hazard ratios. Bootstrap method (1000 replicates) for variation estimation. Total population *N*=25,420. Weighted prevalence. Model 0 unadjusted; model 1 adjusted for sex and age; model 2 additionally adjusted for socioeconomic status, country of birth and chronic disease; model 3 additionally adjusted for leisure-time physical activity, smoking and alcohol consumption; model 4 additionally adjusted for generalised trust in other people. Statistically significant values are shown in bold.

* *p*<0.05; **p<0.01; ***p<0.001.

**Table V.** HRRs with 95% CIs of all-cause, CVD, cancer and other-cause mortality according to visit to both theatre/cinema and arts exhibition, only theatre/cinema or only arts exhibition (active) and no visit (not active).

Cause of death	Model 0		Model 1		Model 2		Model 3		Model 4		Number of deaths
	HR	(95% CI)	HR	(95% CI)	HR	(95% CI)	HR	(95% CI)	HR	(95% CI)	
All causes											1304
Active	1.0		1.0		1.0		1.0		1.0		
Not active	3.2***	(2.8–3.7)	1.9***	(1.6–2.1)	1.7***	(1.5–2.0)	1.4***	(1.2–1.6)	1.3***	(1.2–1.6)	
Cardiovascular disease											383
Active	1.0		1.0		1.0		1.0		1.0		
Not active	3.7***	(2.9–4.8)	2.0***	(1.5–2.6)	1.8***	(1.4–2.4)	1.3*	(1.0–1.8)	1.4*	(1.0–1.8)	
Cancer											517
Active	1.0		1.0		1.0		1.0		1.0		
Not active	2.3***	(1.9–2.9)	1.4**	(1.1–1.8)	1.3**	(1.1–1.7)	1.2	(1.0–1.5)	1.2	(1.0–1.5)	
Others											404
Active	1.0		1.0		1.0		1.0		1.0		
Not active	4.1***	(3.2–5.3)	2.4***	(1.9–3.1)	2.1***	(1.6–2.7)	1.6**	(1.2–2.1)	1.6**	(1.2–2.1)	

The 2008–2016 Scania Public Health Survey with 8.3-year follow-up. Men and women combined. Total population *N*=25,420. Weighted prevalence. Weighted hazard ratios. Bootstrap method (1000 replicates) for variation estimation. Model 0 unadjusted; model 1 adjusted for sex and age; model 2 additionally adjusted for socioeconomic status, country of birth and chronic disease; model 3 additionally adjusted for leisure-time physical activity, daily smoking and alcohol consumption; model 4 additionally adjusted for generalised trust in other people. Statistically significant values are shown in bold.

* *p*<0.05; **p<0.01; ***p<0.001.

## Results

Table I shows that 26.5% had visited both the theatre/cinema and an arts exhibition during the past year, 36.6% had only visited the theatre/cinema, 4.9% had only visited an arts exhibition and 32% had visited neither the theatre/cinema nor an arts exhibition. A significantly higher proportion of men had only visited an arts exhibition or neither the theatre/cinema nor an arts exhibition during the past year. In contrast, a significantly higher proportion of women had visited the theatre/cinema or both the theatre/cinema and an arts exhibition during the past year. Participants with a low SES, born abroad, with chronic disease, with low LTPA, who smoke daily, who abstain from alcohol and with low generalised trust in other people had to a significantly higher extent visited neither the theatre/cinema nor an arts exhibition during the past year. There were also significantly higher proportions of participants with low SES, low LTPA, who smoke daily, who abstain from alcohol and with low generalised trust in other people among those who had only visited the theatre/cinema and only visited an arts exhibition during the past year compared to those who had visited both. There were significantly higher proportions of participants born abroad and participants reporting chronic disease in the category that had only visited an arts exhibition compared to those who had visited both the theatre/cinema and an arts exhibition (Table I). Supplemental Table SI shows that among men, 23.6% had visited both the theatre/cinema and an arts exhibition during the past year, 34.6% only the theatre/cinema, 5.2% only an arts exhibition and 36.6% neither the theatre/cinema nor an arts exhibition, while the corresponding proportions for women were 29.3%, 38.6%, 4.6% and 27.5%, respectively. All variables are displayed by cultural activities participation in Supplemental Table SI.

Table II shows univariate associations between all items/variables and all-cause mortality over the 8.3-year follow-up calculated in survival analyses. Respondents with no visit to the theatre/cinema, with no visit to an arts exhibition, who were men, who were older, who had retired early, who were old-age pensioners, who were on long-term sick-leave, who reported chronic disease, who had low LTPA, who smoked and who had low trust all had significantly higher all-cause mortality than their reference groups. In contrast, respondent groups who had consumed alcohol once a month or more seldom, two to four times a month and two to three times a week displayed significantly lower all-cause mortality than the never consumption reference group. The respondent group born abroad displayed a HRR of 0.9 (95% CI 0.7–1.0).

Table III shows that no theatre/cinema visit during the past year was associated with significantly higher HRRs of all-cause mortality throughout the multiple analyses: HRR 1.3 (95% CI 1.1–1.5) in full model 4 compared to the reference category that visited the theatre/cinema during the past year. Similar patterns with significantly higher HRRs were observed throughout the multiple analyses for CVD mortality: HRR 1.5 (95% CI 1.1–2.1) in model 4 compared to the category that visited the theatre/cinema during the past year. In contrast, the statistically significant association for cancer mortality disappeared after adjustment for health-related behaviours in model 3 (HRR 1.1 (95% CI 0.9–1.4) and for other-cause mortality after adjustments in model 3 (HRR 1.3 (95% CI 1.0–1.8)).

Table IV shows that no visit to an arts exhibition during the past year was associated with significantly higher HRRs of all-cause mortality throughout the multiple analyses: HRR 1.3 (95% CI 1.1–1.5) in full model 4 compared to the reference category that visited an arts exhibition. A similar pattern was observed throughout the multiple analyses for other-cause mortality: HRR 1.6 (95% CI 1.2–2.2) in model 4. In contrast, the statistically significant associations for CVD mortality and cancer mortality disappeared after adjustment for health-related behaviours in model 3: HRR 1.2 (95% CI 0.9–1.6) and HRR 1.2 (95% CI 0.9–1.5), respectively.

Table V shows that the not active category (visit neither the theatre/cinema nor an arts exhibition during the past year) had higher all-cause, CVD and other-cause mortality throughout the multiple adjusted survival analyses in models 0–4 compared to the active (theatre/cinema and arts exhibition or only theatre/cinema or only arts exhibition) reference category. In the final model 4, the not active category displayed a HRR of 1.3 (95% CI 1.2–1.6) for all-cause mortality, 1.4 (95% CI 1.0–1.8) for CVD mortality and 1.6 (95% CI 1.2–2.1) for other-cause mortality. No significant associations between not active and cancer mortality were observed in models 3 and 4.

The Schoenfeld residuals calculated with the active (theatre/cinema and arts exhibition or only theatre/cinema or only arts exhibition) versus not active (neither theatre/cinema nor arts exhibition) categories and all-cause mortality show consistency and stability over the 8.3-year follow-up (Figure 1). The interaction term between active/not active and all-cause mortality was *p*=0.101, which indicates proportionality.

## Discussion

Not visiting the theatre/cinema during the past year was associated with higher all-cause and CVD mortality. No visit to an arts exhibition was associated with higher all-cause and other-cause mortality. The combination of having visited neither the theatre/cinema nor an arts exhibition during the past year was associated with higher all-cause, CVD and other-cause mortality. The lack of significant associations between the two cultural activities and cancer mortality may be due to other aetiology and pathogenesis because the biological mechanisms in the psychosocial stress model specifically apply to CVDs. Tests of proportionality indicated that proportionality criteria were fulfilled.

The findings indicate associations between engaging in arts and culture activities (theatre/cinema and arts exhibition) and mortality, even after adjustments for socio-demographic characteristics, SES, chronic disease, health-related behaviours and trust. These findings are consistent with other studies from the Nordic countries that have shown that engaging in arts and culture activities can have health benefits [18,19]. However, this study showed no association between culture activities and cancer mortality. In contrast to the results of the present study, a previous study found that individuals who engaged in music, singing and theatre had a reduced risk of cancer-related mortality when compared to non-participants [14], and another study found that people living in urban areas who rarely attend cultural activities have a three-fold higher risk of cancer-related mortality when compared to people who are frequent attendees [20]. Yet, this study found an association between attending arts and culture activities and a reduced risk of CVD and other-cause mortality, which resonates with studies showing that an overall reduced risk of CVD mortality was associated with engaging in creative activities [14], and high engagement in cultural activities is independently associated with decreased all-cause mortality [18].

Other studies show that engagement in arts and culture activities can be active ingredients in promoting psychosocial health, including improved psychological well-being and increased social connectedness [9,10,21,22] as well as biological mechanisms such as release of cortisol [23]. The possible causes for associations between people who are participating in arts and culture activities and a reduced risk of cardiovascular and other-cause morbidity might be found in that the positive benefit of arts and culture activities may enhance an existential connection with oneself and the wider world, which can be experienced at different levels. Beyond the psychological, physical and social aspects of existences, they may also promote spiritual and existential health and well-being [24]. In terms of research value, this article adds to existing knowledge of how participation in arts and culture activities can improve different aspect of health and chronic diseases, particularly with a focus on CVD morbidity and other-cause mortality of the population in southern Sweden. This suggests a potential for using arts and culture activities as preventive health measures and in public health promotion.

It is possible in these data to study the cultural activities in relation to specific causes (diagnoses) of death. However, the smaller numbers increase the random error. We identified several specific diagnoses with sufficiently high numbers of deaths to be analysed separately in the present study population (*N*=25,420). We identified them only from the CVD and other-cause categories because the cancer category was not significantly associated with mortality. These specific diagnoses included ischemic heart disease (I20–I25; *n*=169), stroke (I60–I69; *n*=70), pneumonia and influenza (J10–J18; *n*=15), chronic obstructive pulmonary disease (J44; *n*=51), accidents (V01–X59; *n*=37) and intentional self-harm (X60–X84; *n*=24). When we analyse these specific diagnoses in the same multiple survival (Cox regression) models, we find all associations pointing towards increased mortality risk for the group visiting neither the theatre/cinema nor an arts exhibition during the past year. Several of the effect measures are comparatively high, but all are statistically not significant in the final models possibly due to small numbers (Supplemental Table SII). Autopsies were conducted in only approximately 15% of the deaths (see Supplemental Table SIII). In sum, inclusion of only autopsy-verified specific diagnoses would have yielded even smaller numbers and higher random error than the already wide CIs displayed.

### Strengths and limitations

This study is large, population based and longitudinal. The participant population in the 2008 baseline public health survey is representative of the adult population in Scania in 2008 regarding age, sex and education to an acceptable extent. The risk of selection bias is thus moderate [25].

The social participation item has been utilised in Sweden since the 1970s [26,27]. The fact that the numbers of visits to the theatre/cinema and an arts exhibition are not included in these cultural activity items is a limitation of the study. The cultural activity items do not measure frequency. Still, the difference between no visit and one visit is probably more crucial than the difference between one and two or more visits. Swedish register data regarding causes of death have high validity, although cancer diagnoses may have higher validity than other diagnoses. The three aggregate groups of diagnoses are also very broad, and one of them (cancer) most probably has higher validity, which means that the risk of misclassification is smaller than if specific diagnoses were analysed. Also, as demonstrated in the discussion above, analyses of specific diagnoses would incur low statistical power in this questionnaire-based study. It should also be noted that the register data analysed in this study are the same cause of death data as the data from Dödsorsaksregistret analysed in other Swedish register-based studies. The self-reported chronic disease item was included to adjust for chronic disease issues at baseline. This item measures a combination of undiagnosed self-perception of illness and known diagnoses with consequences for perceived health, which may be associated with participation in cultural activities. This item is also associated with all-cause mortality (see Table II), that is, it is a confounder. Unfortunately, we currently do not have access to prescription data or morbidity data (diagnoses) to investigate validity of the chronic disease item. Relevant covariates and mediators were included in the multiple regression analyses. SES can be defined according to occupation, education and income. The three SES dimensions are strongly correlated but not identical dimensions of social status. In this study, SES was measured as occupation and relation to the labour market because income is not available in the data, and education introduces a substantially higher number of internally missing in the analyses. Analyses including education did not alter any of the estimates. Country of birth was included because there are people from 183 countries living in Scania and because country of birth is associated with self-rated health, which is a predictor of all-cause mortality [28].

The exclusion of respondents with internally missing values on any of the items included in this study is preferable to other alternative strategies. The alternatives imputation or letting the number of internally missing increase in each model after the introduction of new and additional covariates in the multiple regression models are methodologically more problematic. Since the other-cause group includes unnatural deaths such as accidents and suicides, the negative association with going to an arts exhibition might be due to case selection. Yet, case selection would then apply to both an arts exhibition and the theatre/cinema and is therefore less likely.

## Conclusions

No visit to the theatre/cinema during the past year was associated with higher all-cause and CVD mortality. No visit to an arts exhibition was associated with higher all-cause and other-cause mortality. The combination of having visited neither the theatre/cinema nor an arts exhibition during the past year was associated with higher all-cause, CVD and other-cause mortality. There is an association between attending arts and culture activities and a reduced risk of CVD and other-cause mortality, although model imperfections are possible.

## Supplemental Material

sj-docx-1-sjp-10.1177_14034948231165853 – Supplemental material for Arts and culture engagement and mortality: A population-based prospective cohort study Supplemental material, sj-docx-1-sjp-10.1177_14034948231165853 for Arts and culture engagement and mortality: A population-based prospective cohort study by Anita Jensen, Mirnabi Pirouzifard and Martin Lindström in Scandinavian Journal of Public Health
