# Supplementary material for: Arts and culture engagement and mortality: A population-based prospective cohort study
Source: Scand J Public Health. 2023 Apr 22;52(4):511–20. doi: 10.1177/14034948231165853 (PMC11179309; doi:10.1177/14034948231165853)
Supplement: sj-docx-1-sjp-10.1177_14034948231165853 – Supplemental material for Arts and culture engagement and mortality: A population-based prospective cohort study [file sj-docx-1-sjp-10.1177_14034948231165853.docx]

| **Table S1.** Descriptive characteristics (%) of age, sex, SES, country of birth, chronic disease, low leisure-time physical activity, smoking, alcohol consumption, and generalized trust in other people by social participation.  The 2008-2016 Public Health Survey of Scania, Sweden. Total population **n=25 420**. **Weighted prevalence.** | | | | | | | |
| --- | --- | --- | --- | --- | --- | --- | --- |
|  |  |  | | | |  | |
|  |  | **(Social Participation)** | | | |  | |
| **Both theatre/**  **cinema and arts exhibition** | **Only theatre/ cinema** | **Only arts exhibition** | **Neither theatre/ cinema nor arts exhibition** | p-value | |  |  |
|  |  | n = 7036 | n = 8938 | n = 1350 | n = 8096 |  | |
|  |  | 26.5% | 36.6% | 4.9% | 32.0% |  | |
|  |  |  |  |  |  |  | |
| **Age**, yrs: mean ±SD ^a^ | | 46.1±16.3 | 40.3 ± 15.9 | 54.4± 15.0 | 50.8 ± 16.4 | <0.001 | |
|  |  |  |  |  |  |  | |
| **Sex^b^** | |  |  |  |  | <0.001 | |
|  | Male | 23.6 | 34.6 | 5.2 | 36.6 |  | |
|  | Female | 29.3 | 38.6 | 4.6 | 27.5 |  | |
| **Socioeconomic status (SES)** ^b^ | |  |  |  |  | <0.001 | |
|  | High non-manual | 48.8 | 34.1 | 4.8 | 12.3 |  | |
|  | Medium non-manual | 38.1 | 40.6 | 4.9 | 16.4 |  | |
|  | Low non-manual | 25.2 | 45.9 | 4.3 | 24.6 |  | |
|  | Skilled manual | 14.5 | 46.1 | 3.3 | 36.1 |  | |
|  | Unskilled manual | 15.4 | 42.1 | 2.6 | 39.9 |  | |
|  | Self-employed/farmer | 29.5 | 35.8 | 5.3 | 29.4 |  | |
|  | Early retired | 13.1 | 18.2 | 6.8 | 61.9 |  | |
|  | Unemployed | 17.7 | 33.1 | 2.7 | 46.5 |  | |
|  | Student | 32.0 | 45.1 | 2.4 | 20.5 |  | |
|  | Old age pensioner | 25.8 | 19.2 | 9.1 | 45.9 |  | |
|  | Unclassified | 20.1 | 46.6 | 3.9 | 29.3 |  | |
|  | Long-term sickleave | 11.6 | 25.4 | 6.4 | 56.6 |  | |
|  |  |  |  |  |  |  | |
| **Country of birth^b^** | |  |  |  |  | <0.001 | |
| Born Sweden | | 28.2 | 38.0 | 4.9 | 28.9 |  | |
| Born abroad | | 18.6 | 30.0 | 4.8 | 46.6 |  | |
|  | |  |  |  |  |  | |
| **Chronic disease^b^** | |  |  |  |  | <0.001 | |
| Yes | | 23.0 | 30.5 | 5.9 | 40.6 |  | |
| No | | 27.8 | 39.0 | 4.5 | 28.7 |  | |
|  | |  |  |  |  |  | |
| **Leisure-time physical activity^b^**  High | | 28.8 | 37.4 | 5.0 | 28.8 | <0.001 | |
| Low | | 12.0 | 31.6 | 4.3 | 52.1 |  | |
|  | |  |  |  |  |  | |
| **Smoking ^b^** | |  |  |  |  | <0.001 | |
| Daily | | 14.9 | 32.0 | 5.0 | 48.1 |  | |
| Yes, but not daily | | 31.2 | 38.2 | 5.3 | 25.3 |  | |
| No | | 28.2 | 37.4 | 4.8 | 29.6 |  | |
|  | |  |  |  |  |  | |
| **Alcohol drinking past year^b^** | |  |  |  |  | <0.001 | |
| Never | | 11.3 | 26.8 | 4.2 | 57.7 |  | |
| Once a month or more seldom | | 16.5 | 40.4 | 4.3 | 38.8 |  |  |
| 2-4 times a month | | 27.8 | 42.8 | 3.7 | 25.7 |  |  |
| 2-3 times a week | | 38.3 | 33.0 | 6.3 | 22.4 |  |  |
| At least 4 times a week | | 38.7 | 20.6 | 9.2 | 31.5 |  |  |
| **Generalized trust in other people^b^** | |  |  |  |  |  | <0.001 |
| High trust | | 30.9 | 36.2 | 5.3 | 27.6 |  |  |
| Low trust | | 18.9 | 37.2 | 4.2 | 39.7 |  |  |
|  | | | | | | | |
| ^a^ p-value: Independent samples ANOVA-test, 2-tailed | | | | | | | |
| ^b^ p-value: Pearson Chi Square test, 2-sided.  The values in parentheses are 95% confidence intervals for mean or percent based on bootstrap method with 1000 number of replicates. | | | | | | | |

| **Table S2.** Hazard rate ratios (HRRs) with 95% confidence intervals (95% CIs) of all-cause mortality and some diagnoses of CVD and other cause mortality according to theatre/cinema and/or arts exhibition visit at least once compared to no visit during the past year.  The 2008 -2016 Scania public health survey with 8.3 years follow-up.  Men and women combined. Total population **n=25 420**. **Weighted prevalence.** | | | | | | | | | | | |
| --- | --- | --- | --- | --- | --- | --- | --- | --- | --- | --- | --- |
|  | **Model 0** | | **Model 1** | | **Model 2** | | **Model 3** | | **Model 4** | |  |
| **Cause of death** | **HR** | **(95%CI)** | **HR** | **(95%CI)** | **HR** | **(95%CI)** | **HR** | **(95%CI)** | **HR** | **(95% CI)** | **Number of**  **deaths** |
| **Ischemic Heart Disease** |  |  |  |  |  |  |  |  |  |  | **169** |
| Visit | 1.0 |  | 1.0 |  | 1.0 |  | 1.0 |  | 1.0 |  |  |
| No visit | **3.7***** | (2.5-5.4) | **2.0**** | (1.3-3.0) | **1.8**** | (1.2-2.8) | 1.2 | (0.8-1.9) | 1.2 | (0.8-1.9) |  |
| **Stroke** |  |  |  |  |  |  |  |  |  |  | **70** |
| Visit | 1.0 |  | 1.0 |  | 1.0 |  | 1.0 |  | 1.0 |  |  |
| No visit | **2.8***** | (1.6-5.0) | 1.5 | (0.8-2.6) | 1.5 | (0.8-2.7) | 1.3 | (0.7-2.5) | 1.2 | (0.6-2.3) |  |
| **Other CVD** |  |  |  |  |  |  |  |  |  |  | **144** |
| Visit | 1.0 |  | 1.0 |  | 1.0 |  | 1.0 |  | 1.0 |  |  |
| No visit | **4.4***** | (2.9-6.7) | **2.3***** | (1.5-3.6) | **2.1***** | (1.4-3.2) | **1.7*** | (1.0-2.8) | **1.7*** | (1.0-2.8) |  |
| **Pneumonia and influenza** |  |  |  |  |  |  |  |  |  |  | **15** |
| Visit | 1.0 |  | 1.0 |  | 1.0 |  | 1.0 |  | 1.0 |  |  |
| No visit | 5.7 | (0.4-89.8) | 3.5 | (0.2-62.4) | 2.8 | (0.2-51.1) | 1.9 | (0.1-37.8) | 1.7 | (0.1-34.0) |  |
| **Chronic obstructive pulmonary disease** |  |  |  |  |  |  |  |  |  |  | **51** |
| Visit | 1.0 |  | 1.0 |  | 1.0 |  | 1.0 |  | 1.0 |  |  |
| No visit | **8.8***** | (3.4-22.7) | **5.2**** | (1.9-14.0) | **4.8**** | (1.7-13.8) | 2.8 | (0.9-8.5) | 2.9 | (0.9-9.0) |  |
| **Accidents** |  |  |  |  |  |  |  |  |  |  | **37** |
| Visit | 1.0 |  | 1.0 |  | 1.0 |  | 1.0 |  | 1.0 |  |  |
| No visit | **2.8*** | (1.3-6.2) | 1.8 | (0.8-4.1) | 1.7 | (0.7-3.8) | 1.4 | (0.6-3.1) | 1.3 | (0.6-2.9) |  |
| **Intentional self-harm** |  |  |  |  |  |  |  |  |  |  | **24** |
| Visit | 1.0 |  | 1.0 |  | 1.0 |  | 1.0 |  | 1.0 |  |  |
| No visit | **3.2*** | (1.0-9.6) | 2.2 | (0.8-6.5) | 2.0 | (0.7-6.0) | 2.2 | (0.7-7.4) | 2.5 | (0.7-8.5) |  |
| **Other PCAI** |  |  |  |  |  |  |  |  |  |  | **277** |
| Visit | 1.0 |  | 1.0 |  | 1.0 |  | 1.0 |  | 1.0 |  |  |
| No visit | **3.9***** | (2.9-5.3) | **2.2***** | (1.6-3.1) | **1.9***** | (1.4-2.7) | **1.5*** | (1.1-2.1) | **1.5*** | (1.1-2.1) |  |
|  |  |  |  |  |  |  |  |  |  |  |  |
| Model 0 unadjusted. Model 1 adjusted for sex and age. Model 2 additionally adjusted for socioeconomic status, country of birth and chronic disease. | | | | | | | | | | | |
| Model 3 additionally adjusted for leisure-time physical activity, daily smoking and alcohol consumption. Model 4 additionally adjusted for generalized trust in other people. | | | | | | | | | | | |
| Significance levels: * p<0.05, ** p<0.01, *** p<0.001. Weighted Hazard Ratios. Bootstrap method (1000 replicates) for variation estimation. | | | | | | | | | | | |

| **Table S3.** Number of autopsies. The 2008 -2016 Scania public health survey with 8.3 years follow-up. Men and women combined. Total population **n=25 420**. | | | |
| --- | --- | --- | --- |
|  | **Clinical autopsy** | **Extended forensic autopsy** | **Forensic autopsy** |
| **Ischemic Heart Disease** | 48 | 1 | 23 |
| **Stroke** | 4 | --- | 2 |
| **Pneumonia and influenza** | --- | --- | 4 |
| **Chronic obstructive pulmonary disease** | 3 | --- | --- |
| **Accidents** | 1 | --- | 22 |
| **Intentional self-harm** | --- | --- | 24 |
